# Nanophysics Is Boosting Nanotechnology for Clean Renewable Energy

**DOI:** 10.3390/ma17215356

**Published:** 2024-11-01

**Authors:** Rui F. M. Lobo, César A. C. Sequeira

**Affiliations:** 1 Laboratory of Nanophysics/Nanotechnology and Energy (N2E), Center of Technology and Systems (CTS), Physics Department, NOVA School of Science & Technology, NOVA University of Lisbon, 2829-516 Caparica, Portugal; 2 Materials Electrochemistry Group, Department of Chemical Engineering, Instituto Superior Técnico, Av. Rovisco Pais, 1049-001 Lisboa, Portugal

**Keywords:** clean energy, energy storage, green hydrogen, nanocatalysts, laser and plasma processing, ultramicroscopy

## Abstract

As nanophysics constitutes the scientific core of nanotechnology, it has a decisive potential for advancing clean renewable energy applications. Starting with a brief foray into the realms of nanophysics’ potential, this review manuscript is expected to contribute to understanding why and how this science’s eruption is leading to nanotechnological innovations impacting the clean renewable energy economy. Many environmentally friendly energy sources are considered clean since they produce minimal pollution and greenhouse gas emissions; however, not all are renewable. This manuscript focuses on experimental achievements where nanophysics helps reduce the operating costs of clean renewable energy by improving efficiency indicators, thereby ensuring energy sustainability. Improving material properties at the nanoscale, increasing the active surface areas of reactants, achieving precise control of the physical properties of nano-objects, and using advanced nanoscale characterization techniques are the subject of this in-depth analysis. This will allow the reader to understand how nanomaterials can be engineered with specific applications in clean energy technologies. A special emphasis is placed on the role of such signs of progress in hydrogen production and clean storage methods, as green hydrogen technologies are unavoidable in the current panorama of energy sustainability.

## 1. Introduction

Although this review is not intended to focus on physics, but rather on its application to energy materials, it is worth mentioning that nanophysics studies physical phenomena at the nanoscale and properties that emerge at such small sizes. It forms the background of nanotechnology by enabling precise control over the behavior of matter at this scale. Unlikely chemistry, which focuses on “blind molar transformations”, and materials science, which looks at the macroscopic properties of materials, nanophysics centers on the fundamental phenomena that govern behavior at the atomic and molecular levels.

In his famous lecture “Plenty of Room at the Bottom”, Feynman considered the possibility of the direct manipulation of individual nano-objects, even atoms, and reckoned that there would be no impediment to doing this based on the laws of physics. Still, the right tools were lacking then [1]. Although the electron microscope was subsequently improved, it was necessary to wait at least two more decades for such an aim to be realized thanks to the rapid development of ultramicroscopy with near-scanning probes, among which the Scanning Tunneling Microscopy (STM) and Atomic Force Microscopy (AFM) made it possible to take the decisive steps to enter the Nano era [2,3,4].

This feat opened a new imaging panorama and provided new tools to implement the “observe–modify–observe” cycle, an analogy with what had happened earlier with the development of optical microscopy. Many powerful physical techniques that act at the atomic, molecular, and nanoscales, developed throughout the 20th century, could also play a decisive role in the “modify” stage of the mentioned cycle [5,6]. In addition, we should also mention the significant progress that chemistry made, especially in the fields of catalysis, analysis, electrochemistry, and photochemistry [7].

As usual, advances in physics and chemistry lead to disruptive innovations in material technologies, and in this case, nanostructured and nanocomposite materials emerged [8]. They can be tailored so that through functionalization and the assembly of nano-objects, these objects can give rise to required or even innovative properties [4,9,10]. With nanoparticles as components, they can be designed using new top–down or bottom–up techniques and, in addition to the fact that quantum effects cannot be ignored at the nanoscale, the consequences of increasing the surface/volume ratio must also be considered, which has led to looking for nano-objects with a total absence of defects. In this vein, there has been a fascination throughout history for perfect geometrical shapes with high symmetries. This time, one class of atomic clusters formed only with the magnificent carbon atoms emerged: the fullerenes [11]. C_60_ is the most famous cluster among them. They are artificially produced using laser vaporization of graphite, being the as-formed plasma carried away by a supersonic expansion helium beam [12]. In the experiment, other carbon clusters were formed that ranged in molecular masses, but C_60_ and C_70_ were predominant, and C_60_ was subsequently recognized in some other experiments as a highly symmetric molecular nano-object displaying a regular truncated cage-like icosahedron [12,13,14].

Mass spectrometry combined with ultramicroscopy and X-ray analysis techniques shows consistency with arrays of spherical C_60_ molecules, approximately one nanometer in van der Waals diameter. If the soot is produced by the pyrolysis of aromatic hydrocarbons instead of the laser ablation of graphite, Soxhlet extraction from the soot (with organic solvents) will be required, followed by chromatographic separation. This illustrates a situation where physical or chemical methods can be applied to generate cage-like nano-objects. It is worth noting that such an alternative has been repeated in many subsequent experiments, which has led to obtaining other cage-like nanocarbons, particularly nanotubes and endohedral (or exohedral) fullerenes/nanotubes [4,9,10]. It is important to remember that the synthesis of nanomaterials begins with the nano-synthesis of essential components that may serve as fundamental building blocks in nanoscience [15]. Thus, the methods to produce fullerenes and carbon nanotubes can involve more or less chemicals, which have further consequences in contamination and purification costs. The quest for the most efficient and environmentally friendly method of producing fullerenes and carbon nanotubes is ongoing, with researchers continually striving to minimize contamination and purification costs. Environment-friendly synthesis routes that utilize fewer chemicals (or rely on renewable resources) and develop efficient catalytic processes that enhance selectivity (and yield) while reducing the need for excess chemicals are the main goals to pursue. In this aim, gas phase synthesis techniques (such as laser ablation, laser pyrolysis, and arc discharge) allow the production of high-purity fullerenes and carbon nanotubes directly from carbon-containing gasses. The optimization of these processes could minimize contamination from precursor materials, develop continuous production methods, enhance efficiency, and reduce the need for batch processing, thereby minimizing the accumulation of impurities over time [4,9,10,15].

Solvent-based methods (such as liquid-phase exfoliation and chemical reduction) typically involve dissolving a carbon source (e.g., graphite) in a solvent and then using chemical reactions or physical processes to isolate fullerenes. While these methods are relatively simple and versatile, they may result in lower purity and require extensive purification. In turn, Chemical Vapor Deposition (CVD) involves the decomposition of hydrocarbon precursors at high temperatures in the presence of a catalyst to form carbon nanotubes or fullerenes. While CVD offers excellent scalability and can produce high-quality nanocarbons, it often requires additional purification steps due to the formation of carbonaceous byproducts. Thus, CVD and solvent-based approaches are more scalable but may require further purification to achieve the desired purity level. On the contrary, physical methods involving plasma intermediation, like laser ablation, laser pyrolysis, or arc discharge, tend to offer higher purity, although they can be more energy-intensive and expensive.

**Figure 1:**
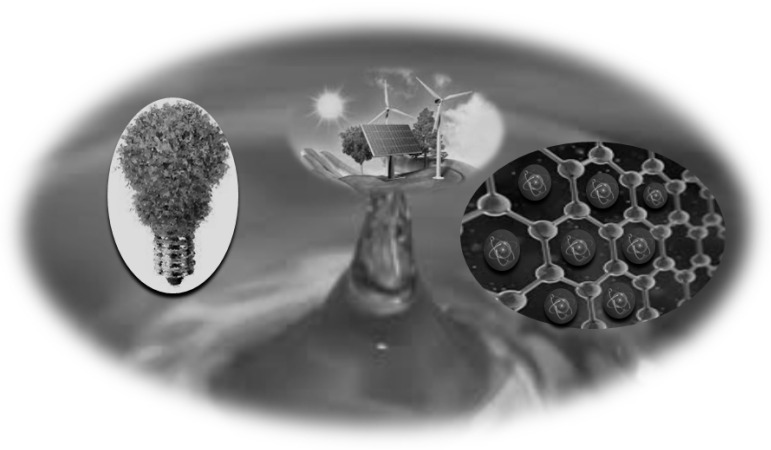
Graphical representation of the nanoworld for clean energy [adapted and assembled from the internet].

Clean energy and renewable energy are closely related concepts, but not equivalent. Clean energy encompasses energy sources and technologies with minimal environmental impact that produce low or zero emissions of pollutants and greenhouse gasses (see a graphical representation in Figure 1). Hydrogen can be considered a clean energy source, particularly when produced through renewable methods, although it is not inherently renewable. Hydrogen can be produced through various methods, some clean and renewable, while others are not. Electrolysis is a clean method of producing hydrogen by splitting water molecules into hydrogen and oxygen using electricity, often provided by renewable sources like solar or wind power. In turn, Steam Methane Reforming (the most common method of hydrogen production today), relies on fossil fuels, primarily natural gas, and produces carbon dioxide emissions as a byproduct. Thus, in this case, carbon capture and storage technologies must be further improved to become cleaner processes. Methods of obtaining hydrogen using precursors (like hydrocarbons, ammonia, or biomass) also need additional byproduct elimination steps, so they are not truly clean. When hydrogen is used as a fuel, it emits only water vapor and heat, making it a clean energy carrier. It can be used in fuel cells to produce electricity for various applications, including transportation, heating, and industrial processes. Therefore, hydrogen plays a significant role in the transition to cleaner and more sustainable energy systems when produced and utilized in an environmentally responsible manner.

Nanophysics enables the understanding and manipulation of material properties at the nanoscale. By designing and synthesizing nanomaterials with specific properties, such as enhanced light absorption, improved conductivity, and higher catalytic activity, nanotechnology can create more efficient solar cells, batteries, and catalysts for renewable energy generation and storage. Nanotechnology achievements have led to energy devices with improved performance and functionality. For example, the design of nanostructured electrodes for batteries and supercapacitors, as well as nanostructured photovoltaic materials for solar cells, leads to higher energy density, faster-charging rates, and a longer life cycle.

Additionally, nanophysics provides advanced characterization techniques which allow researchers to study structural, electronic, and optical nanomaterials properties, with unprecedented resolution and sensitivity. These techniques, such as scanning tunneling microscopy, atomic force microscopy, and transmission electron microscopy, enable researchers to deeply understand the fundamental mechanisms governing the behavior of nanomaterials and optimize their performance for renewable energy applications. The synergy between nanophysics and nanotechnology holds great promise for revolutionizing renewable energy technologies by enabling the development of more efficient, cost-effective, and sustainable energy solutions.

Attention has been devoted to the role of nanocarbons in innovative clean energy technologies with special emphasis on renewable hydrogen. They display unique properties that enhance hydrogen production, storage, and utilization efficiencies. Cage-like nanocarbons assume several morphologies and assemblies that in any case display a network of nanoporosity. This aspect is of valuable importance because it favors the increase in internal and external global active surface areas available for reactions, which critically influences the kinetic efficiency and catalytic performances.

It is appropriate to mention that cage-like chemical compounds, called metal–organic frameworks (MOFs), have also gained significant attention, namely in water splitting and hydrogen storage improvement performances. They also allow for the incorporation of different metal centers, enhancing the number of active sites for catalysis. However, they commonly require synthesis procedures involving expensive precursors, limiting their large-scale applicability. In addition, MOF hydrogen storage capacities at pressure and ambient temperatures are still a challenge today [16,17]. In any case, the benefits of using nanomaterials are unquestionable, making the privileged choice of storage in solid media irreversible [16,17,18,19].

Doped nanocarbons promise many favorable characteristics over single precious metal catalysts: earth-abundance, better prices, a higher degree of specific surface areas and pore volumes, larger amounts of surface defects, stronger tolerance to acidic or alkaline environments, and sustainability [20].

Endohedral fullerenes exhibit unique and fascinating chemical characteristics that may differ substantially from those of the encapsulated atom or molecule as well as the fullerene itself. NMR spectroscopy has been used to track the motion of the encapsulated atoms, which have been observed to move in circles inside the C_60_ cage [21].

From everything that has already been said, it can be concluded that carbon nanotechnology driven by theoretical and experimental nanophysics could open new horizons for clean energy applications based on 3D cage-like nano-objects. Given this reality, this topic will be given a prominent place in this review.

## 2. Nano-Objects for Clean Energy

In the realm of clean energy efficiency, carbon nanostructures have shown great promise in various applications. They hold the potential to effectively accelerate the transition towards a cleaner and more sustainable energy future. To provide a logical chain from now on, let us illustrate the progress made in cage-like nanocarbons applied to high-temperature superconductivity, which is one of the “Grails” in the aim of energy. The synthesis of a carbon crystal formed by several C_60_ molecules is called fullerite and when doped, led to the discovery of a new family of high-temperature superconductors. Roughly, fullerite at 230 Kelvin has a face-centered cubic (fcc) lattice dense packing of molecules, with 0.1 nm separation between molecular centers; at lower temperatures, the lattice changes to a simple cubic lattice. NMR and neutron diffraction fullerite studies concluded that the C_60_ molecules display rotational motion. Furthermore, fullerite is dielectric but can be doped with alkali atoms leading these elements to form interstitial alloys (thus contributing electrons to the lowest energy empty band) and becoming a conductor. For example, the crystal structures of M_6_C_60_ (M = K, Rb, Cs) are body-centered cubic (bcc) with the alkali atoms occupying the quasi-tetrahedral interstitial sites, and with the M valence electron transferred to the C_60_. Furthermore, M_3_C_60_ has an fcc packing with the M atoms occupying both the octahedral or tetrahedral interstitial sites. The M_3_C_60_ crystals are metallic and display a high superconducting critical temperature. Hole-doped fullerite instead of electron-doped is also prone to display high-temperature superconductivity [22]. The preparation of superconducting alkali fulleride films using molecular beam epitaxy (MBE) and corresponding analysis through cryogenic scanning tunneling microscopy (STM) results can contribute to the study of the physical superconducting mechanism involved, pointing to the traditional electron–phonon interaction [23].

While carbon nanostructures themselves are not superconducting at room temperatures, they have been incorporated into hybrid materials to enhance their superconducting properties. For example, carbon nanotubes and graphene have been used as additives or substrates in superconductor composites to improve critical current density, enhance flux pinning, and increase the transition temperature of superconducting materials. These advancements could enable more efficient energy transmission and storage systems.

Since endohedral fullerenes may have an atom or a molecule trapped inside the hollow cage-like structure made of carbon atoms, they have a great potential application across the clean energy field, namely in catalysis and storage. The encapsulated atom or cluster of atoms within the fullerene cage can alter the electronic structure of the fullerene, leading to unique catalytic properties. This altered electronic environment can influence the adsorption, activation, and transformation of reactant molecules on the surface of the fullerene, thereby affecting the catalytic activity and selectivity [24]. In addition, the electronic properties of endohedral fullerenes can be tuned by varying the encapsulated species or by functionalizing the fullerene cage. Also, the large surface area displayed by the hollow cage provides many active sites where catalytic reactions are likely to occur. This high surface area can enhance the efficiency of catalytic processes by increasing the contact between reactant molecules and the catalyst. The hollow cage ensures additional stability of the nanocatalyst, which can be maintained over multiple reaction cycles [24]. One concrete example of the importance of endohedral fullerenes in catalysis is their potential application in hydrogenation reactions. Hydrogenation can be integrated with carbon capture utilization and storage technology (CCUS), and traditionally it is catalyzed by transition metals such as Pt, Pd, or Ni. However, these catalysts often suffer from limitations such as high cost, limited selectivity, and susceptibility to poisoning by reaction intermediates [25].

In hydrogenation reaction catalysis, endohedral fullerenes are magic due to their unique electronic and structural properties. For example, endohedral fullerenes containing metal atoms such as titanium, lanthanides, or alkali metals have been studied for their catalytic activity in hydrogenation reactions.

In turn, nanostructured fullerene layers can be utilized in energy storage devices such as supercapacitors and batteries. Both increased surface area and optimized charge transfer can enhance energy storage capacity and cycling stability. Nanostructuring the fullerene C_60_ beam deposited films is achieved by electrochemical reduction in KOH aqueous solution [26]. Scanning tunneling microscopy (STM), fluorescence spectroscopy, cyclic voltammetry, x-ray diffraction, and scanning near-field optical microscopy have all demonstrated that alkali fulleride clusters are formed at the electrode. These methods yield results that are consistent with the interpretation of the nanometer-sized fulleride cluster. Specifically, when film thickness increases and aggregation becomes more efficient, the fluorophore fluorescence lifetime diminishes. Single buckyballs are not visible in the STM image of the fullerene upper layer obtained after the film reduction for the thicker film; instead, clusters of likely K_3_C_60_ molecules are seen. A notable alteration in the film structure was noted in the thicker films. The C_60_^−^ anion is produced when a C_60_ molecule receives its first electron injected into it. There is an irreversible component to the reaction. The reaction is accompanied by an irreversible intercalation of K^+^ cations into the lattice of molecular fullerene crystals to compensate for the negative charge. The fullerene reduction was accompanied by an aggregation process in which clusters could be formed [26].

Nanostructured fullerene layers are also utilized in photoelectrochemical cells for solar energy conversion. The enhanced charge transfer properties and surface area facilitate efficient charge separation and collection, which improves device performance. In particular, the influence of nanocarbons in perovskite solar cells is well documented [27].

Therefore, the electrochemical nanostructuring of fullerene layers offers opportunities to tailor the properties of electrode materials for a wide range of applications in energy storage, sensing, catalysis, and optoelectronics. In catalysis, the nanostructured fullerene layers can serve as efficient catalysts for various electrochemical reactions, including oxygen reduction reactions (ORRs) and hydrogen evolution reactions (HERs). The suitable control of the nanostructuring enables tuning the catalytic activity and selectivity of these materials [28].

One must remember that nanophysics has provided advanced characterization techniques that allow researchers to deeply study nanomaterials’ structural, electronic, and optical properties with unprecedented resolution and sensitivity (STM, AFM, TEM). Nanotechnology enables the development of novel energy conversion and storage devices with improved performance and functionality. For example, nanophysics allows the design of nanostructured electrodes for batteries and supercapacitors and nanostructured photovoltaic materials for solar cells, leading to higher energy density, faster charging rates, and a longer life cycle.

In energy conversion and storage, nanocarbons are integral components in devices like lithium-ion batteries and supercapacitors. By incorporating nanocarbons into these energy storage systems, researchers aim to improve their energy density, cycling stability, and charging/discharging rates, thereby enabling more efficient and reliable energy storage solutions; this includes, as we will see later, applications for renewable hydrogen technologies. The remarkable improvements in energy storage capacity, charging rates, and cycling stability can be attributed to the large surface area and excellent electrical conductivity of carbon nanotubes. In lithium-ion batteries, for instance, graphene-based electrodes performed better and allowed for quicker charging and a longer lifespan. The unique mechanical, physical, chemical, and electronic properties of carbon nanotubes (CNTs) have also drawn a lot of attention. These properties present intriguing opportunities for the fabrication of supercapacitors [29], field emission-based devices [30], gas and flow sensors [31,32], and hydrogen storage [33]. CNTs may serve as genuine precursors of perfect materials. They have a high Young’s modulus, high porosity, low reactive, high electrical and thermal conductivities, are light, flawless, and exhibit remarkable resistance to fracture. There are several methods to synthesize CNTs, each producing them with different characteristics. Thus, the final application may influence which method is more appropriate. The properties of the nanotubes largely depend on the growth process and some influential parameters like growth time, growth temperature, growth pressure, and catalyst, and its concentrations may be varied systematically to have better control over the nanotube mainly in terms of the diameter, density, and length of the nanotubes [34].

**Figure 2:**
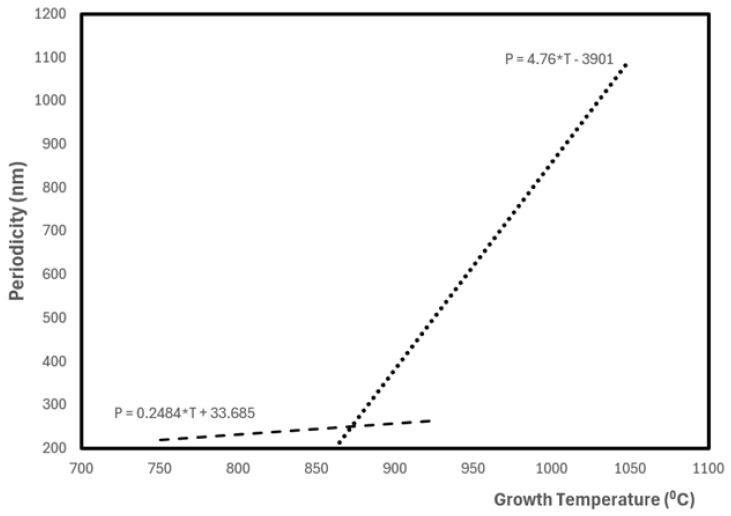
Expected variation in a SWNT spiral with the growth temperature. P and T stand, respectively, for periodicity and temperature [based on experimental information of references cited in the corresponding text].

Plasmas are ideal media for implementing nanophysics transformations and CNTs have been mostly produced through laser ablation or arc discharge techniques. They use the bottom–up strategy principle based on microplasma formation that afterward leads to condensation in a vacuum [4,12]. In both cases, the CNT formation efficiency drastically increases with the incorporation of catalysts. Furthermore, the cold plasma chemical vapor deposition (CPCVD) technique can be applied to grow carbon nanotubes over a metallic wire [35]. In this technique, carbon nanotubes grow directly over the catalyst-supported wire by resistive heating in an acetylene atmosphere above 700 °C. The growth temperature is crucial for the control and selective growth of the nanotubes (nanotube diameter, length, periodicity, and density). Growth temperature plays a significant role in determining the chirality and diameter of SWNTs. Chirality describes the arrangement of carbon atoms in the nanotube wall, which directly influences its electrical and mechanical properties. The catalyst used in SWNT growth (typically transition metals like Fe, Co, or Ni) is also sensitive to temperature and may in addition affect the spiral formation; at higher temperatures, the catalyst is more active and leads to a more controlled growth, mitigating irregularities which contribute to spital formation. Low temperatures favor spirals to form, due to increased defect density and slower, less controlled growth. Higher temperatures encourage more uniform and straighter nanotubes, with less spiraling, due to faster growth and fewer defects. An interesting observed behavior of the nanotubes is their spirality (helical nature variation), as the increase in periodicity of the helix tube is found with temperature [36]. Hexagonal carbon rings are the basic structural units for the graphene shell which form carbon nanotubes. These nanotubes have a structure that is both wavy and helical. Along the helical structure, there are variations in the coiling periodicity. The size of the catalyst has an impact on the spiral structure’s growth root when it is introduced. The large particle’s various crystallographic faces exhibit varying rates of carbon diffusion, which results in the formation of a helical or spiral structure [37]. The helical or spiral structure of the nanotubes is determined by the rate at which the pentagon (P) and heptagon (H) carbon rings are created, in agreement with the kinetically controlled growth model [38]. Whereas the heptagon carbon ring forms a negative curvature, the pentagon carbon ring produces a positive curvature. The pairing of the pentagon–heptagon carbon rings creates the helical or spiral structure of the nanotube, and the spiral periodicity depends upon the adjacent distance between P–H pairs. The limited or lower supply of P–H pairs restricts the nanotube to curling with temperature, leading to the increase in periodicity. It is the consequence of temperature variation influencing the diffusion of carbon atoms through the metal particles. Then, the periodicity increase in the helix tube with increasing temperature is expected (Figure 2). The change in periodicity with the growth temperature is not monotonic but displays two approximate linear regimes. The periodicity remains roughly constant with a growth temperature of up to 870 °C. Then, a great increase is observed above that temperature, and around 1050 °C, it is more than five times that for the spiral nanotubes at low temperatures [38].

Carbon nanotubes of high purity can also be obtained through Laser-Induced Pyrolysis (LIP), which is a technique involving the use of an infrared laser to interact with gas-phase reactants: the plasma plume results in molecular decomposition and forms vapors initiating nucleation, followed by the growth of nanoparticles that fall by gravity [39]. It has the advantage of producing a precise and controllable particle size distribution (PSD) [40,41], without introducing contaminants (as solvents).

The LIP technique in the gaseous/vapor phase is based on the resonance between the emission line of a continuous-wave (CW) CO_2_ laser and the mid-infrared absorption band of at least one gas-phase component, for instance, ethylene. The main vibrational frequency of C_2_H_4_ is its CH_2_ wagging mode, *ν* = 949 cm^−1^; this band has many vibration–rotation lines overlapping the emission lines of the CO_2_ laser near 10.6 μm (944 cm^−1^). Thus, the photon energy from the laser raises the ethylene molecules to higher excitation levels. The vibration–translation relaxation of the system leads to the dissipation of the excited modes translationally and rapidly achieves a high temperature, producing ethylene decomposition. The laser description (itself) can be found elsewhere [42,43], and it is relevant for LIP processing to understand its basic working principle.

Taking advantage of the vibration–rotation bands of the linear triatomic molecule CO_2_, the laser transitions can be adjusted by a tuning element (diffraction grating in the laser cavity). This element can be rotated by a micrometer screw to select a specific rotational line of the vibrational transition in the P and R bands. The control of the CO_2_-pulsed tunable laser performance is achieved following a procedure that optimizes both the power and energy [43]. Combining a pulse mode with the grating tuning facility, the laser can scan the working wavelength between 9.2 and 10.8 μm (operating on more than 60 lines) with repetition rates ranging from a single shot to 200 Hz. The strongest lines correspond to a maximum energy of 50 mJ/pulse. Following a suitable procedure, the energy values can be optimized in intensity and stability, indirectly improving the laser power. Besides energy, power was also measured and improved, following the same procedure. At a repetition rate of 100 Hz and 10.6 mm of wavelength, the power output is around 50 W.

**Figure 3:**
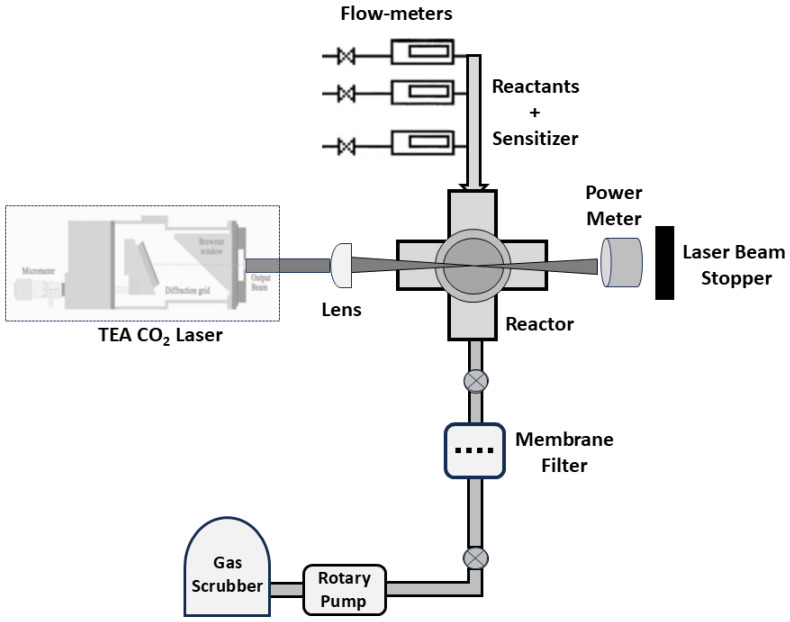
Laser-induced pyrolysis setup.

The LIP process takes place in a cross-shaped flux reactor in the gas phase, and KCl windows are used to ensure transparency to the infrared radiation (setup in Figure 3). Using a ZnSe lens, the laser beam is focused onto the reaction center, resulting in a width of 4 mm (for 100 W this corresponds to a power density of 2 × 10^3^ W/cm^2^). Gas flows are regulated with flowmeters to maintain consistent pressure in the reactor, and an outer coaxial argon flow ensures the confinement of the reactant gas towards the nozzle axis. Upon sudden cooling, these products precipitate in the shape of nanoparticles carried away by the gas flow. To create carbon nanotubes (CNTs), a process using iron pentacarbonyl, Fe (CO)_5_, vapor flow is utilized. The fine iron particles produced from the decomposition of organometallic precursor molecules catalyze the formation of CNTs. Ethylene gas is used as a carrier for Fe (CO)_5_ vapor and directed through a bubbler containing liquid iron pentacarbonyl at room temperature. The mixture (Fe (CO)_5_ vapor + ethylene) is pyrolyzed in the flow reactor due to infrared radiation, which heats the gas phase. Ethylene gas also serves as a sensitizer, activating the laser reaction and speeding up the Fe (CO)_5_ dissociation. Iron nanoparticles, necessary for catalyzing the formation of CNTs, are obtained from the decomposition of Fe (CO)_5_ during the laser-induced reaction [44]. Ethylene has resonant absorption at a wavelength of 10.6 μm and high dissociation energy, which results in internal energy exchange with acetylene molecules that do not absorb the CO_2_ laser radiation, heating the entire gas mixture [45]. The powder obtained (through pyrolysis and incomplete combustion of the carbon precursor) is trapped in the collector’s filter and the soot is weighed. These powders are investigated by ultramicroscopy and display carbon nanotubes with different structures. Some nanotubes come together with iron particles which may have played a role in the catalysis of the nanotube growth [46,47]. Such fine iron particles were produced in the same experiment by decomposing the organometallic precursor molecules [44]. Specific conditions, such as laser parameters, gas composition, and reaction time, were found to influence the morphology of the soot observed by ultramicroscopy. The CO_2_ laser-induced pyrolysis of the gaseous mixture leads to the formation of carbon nano-powders and CNTs, whose relative percentage depends on the specific conditions employed during the process.

The incomparable properties of the individual CNTs enable a range of applications with a special emphasis on the energy domain, ranging from improvements in conventional devices (electronics, sensors, energy storage) to new technologies [48,49]. Techniques to assemble CNTs into threads, fibers, and yarns progressed very fast, thus assuring a solid path to a better understanding and applications [50,51,52].

Dry spinning relies on van der Waals forces between CNTs and uses pristine CNT arrays during thread assembly. In contrast to other techniques for CNT synthesis and fiber processing, dry spinning reduces the number of contaminants from the assembly process. It permits the assembly of longer, uniform-length CNTs (usually half a millimeter long into a thread) without the use of surfactants or acid solvents. Additionally, it will make it possible to assemble threads with uniform diameters by regulating the number of CNTs proportionally spun within the thread to the width of the array. It is possible to assemble high-quality drawable CNT arrays into threads of consistent diameter and to precisely control how many CNTs are included in the thread assembly [53]. Furthermore, spinnable carbon nanotubes (CNTs) are a valuable format for investigating the physical characteristics of carbon nanotube fiber assemblies in their pristine states. This is because they do not require any additional chemicals or solvents for their assembly into thread, are uniform in length, and have a relatively narrow diameter distribution.

Trends in electrical resistivity and mechanical strength that resulted from alterations in the manufacturing parameters have been reported, allowing for changes in the intrinsic physical properties of the assembled material, such as electrical resistivity. It is possible to correlate electrical resistivity and mechanical strength as a function of diameter, density, and turns/meter [54]. The findings indicate that increasing the turns per meter supplied to the CNT array width during thread assembly is what causes the change in electrical conductivity. An association has been observed between the electrical conductivity and the twist angle. The contact area between various CNTs determines changes in electrical resistivity, which is influenced by density and mass per unit length [54].

Intermetallic nanoparticles are another nano-object type with great potential for clean energy applications. For example, TiFe intermetallic nanoparticles (NPs) are promising for increasing hydrogen storage in solid media, and they can provide excellent corrosion resistance, good hydrogen storage, and magnetic properties [55]. One effective technique for creating intermetallic NPs, or ultrafine particles of multiple metals, is the hydrogen plasma–metal reaction [56]. The following is the process by which the NPs are formed from the Ti–Fe binary alloy: First of all, under hydrogen plasma, Fe and Ti vapors form simultaneously, and Ti cannot react with hydrogen in this situation. Second, vapors of Fe and Ti start to condense and solidify. At this point, Ti and Fe atoms collide and condense in the same metal cluster, causing the particles to further grow into nanoparticles. Rare earth oxide nanoparticles are also very promising as nanocatalysts owing to their valence states [57,58].

## 3. Application to Clean Hydrogen Technology

This section presents recent developments in clean hydrogen energy, mainly focused on nanophysics and the related nanotechnology methods that have already been explained. Nanoparticles, fullerenes, carbon nanotubes (CNTs), graphene, and their derivatives play a significant role in innovative renewable hydrogen technologies, due to their unique properties that enhance various aspects of hydrogen production, storage, and utilization. They have proved to be of unquestionable help in several ways. They offer a promising solution for hydrogen storage, addressing one of the major challenges in the widespread adoption of hydrogen as a clean energy carrier [59].

Hydrogen storage in adequate solid media (like innovative solid absorbents) may represent a better choice for storing hydrogen in the face of the more energy-costly common methods, since they show better performances and simultaneously reduce costs and weight. Various metal alloys have been explored and studied in recent decades, with such aims in mind [60]. For example, magnesium nanoparticles have advantages compared with bulk metal since they are lightweight, less costly, and have more nucleation sites for magnesium hydride. When monitoring the hydrogen content versus time (by recording the change in gas pressure in a constant volume), the hydrogen absorption reaches equilibrium. When MgH_2_ is formed with a surrounding Ni catalyst present, the sample expands and disintegrates into smaller particles because of the entry of H atoms, which then could react with Ni particles to form Mg_2_NiH_4_ [61]. The resistance to oxidation of these as-formed nanoparticles decreases when the mean particle diameter decreases, becoming stable below 470 K. Hydrogen absorption curves of the Mg_2_Ni intermetallic compound at different temperatures under a 40 bar hydrogen atmosphere typically show that the gas is quickly absorbed at room temperature. The absorption rate increases with temperature, reaching a saturation value in 15 min at 298 K. The maximum hydrogen storage capacity is about 2.8 wt% at 293 K [61]. Introducing a porous structure with a large surface area opens an additional window to provide ample space for hydrogen adsorption through physisorption or chemisorption [62,63,64]. Functionalized nanocarbons represent a new line of research [65] with great potential to enhance hydrogen adsorption capacities and optimize storage conditions, enabling safe and efficient hydrogen storage [66,67,68].

Regarding membrane technologies for hydrogen purification and separation, graphene-based membranes demonstrate high permeability for hydrogen molecules while blocking other gasses. This enables the selective separation of hydrogen from gas mixtures produced in industrial processes or from renewable sources. These membranes offer a cost-effective and energy-efficient method for hydrogen purification [69,70].

Regarding hydrogen utilization in mobile applications and local small-scale energy production, nanocarbons are ideal electrode materials in fuel cells, particularly proton exchange membrane fuel cells (PEMFCs). Their high electrical conductivity, mechanical strength, and chemical stability make them suitable for manufacturing durable and efficient electrodes. Nanostructured carbon materials can also enhance the electrochemical surface area, facilitating faster hydrogen oxidation and oxygen reduction reactions within the fuel cell, thereby improving its performance and durability [71,72,73,74].

Carbon nanostructures, like graphene and graphane, have also significantly contributed to superconductivity research in clean hydrogen energy. Recently, graphene and borophane revealed promising hydrogen storage nanostructures [75,76]. Graphane is a hydrogenated form of graphene (hydrogen atoms are bonded to the carbon atoms of the graphene lattice) and shows potential in hydrogen storage due to its high theoretical gravimetric hydrogen capacity and stability. On the other hand, borophane (the graphane analog for boron) also has a potential material for hydrogen storage at high densities due to its high hydrogen binding energy and stability. In addition, following the recent discovery [77] of superconductivity at a very high temperature (Tc = 203 K in a sulfur–hydrogen system under pressures ∼100 Gpa), there has been a resurgence of interest in H-containing superconductors, focusing on H-rich bulk systems. Thus, it seems natural that low-dimensional variants of Cooper pairing must be explored in the framework of phonon-mediated superconductivity. So, the development of atom-thin materials provides a suitable medium for studying the 2D limit of the phenomenon. Borophene (the graphene analog to boron) displays a metallic ground state and represents an adequate platform for exploring 2D superconductivity. In addition, recent theoretical studies [78,79,80] have reported an increase in Tc for several 2D materials upon hydrogenation, and consequently p-doped graphene has been postulated to be a high-temperature BCS theory superconductor with a Tc above 90 K [81].

Finally, nanoparticles, fullerenes, carbon nanotubes (CNTs), graphene, and their derivatives act as excellent catalysts for hydrogen production in processes such as water electrolysis, photocatalytic water splitting, or hydrocarbon conversion. Their high surface area and electronic properties make them efficient catalysts, accelerating the chemical reactions involved in hydrogen generation. In this regard, it is now opportune to consider how nanophysics underpins the interpretation of these results and allows future developments to be accelerated. Factors affecting the catalytic properties of nanoparticles are their sizes, shapes, size distributions, spatial dispersion in the preparation medium, and, of course, the reaction thermodynamic conditions.

When a solid surface can serve as a catalyst, one often distinguishes between the so-called Eley–Rideal mechanism, where the molecules from the gas react with surface chemisorbed reagents, and the Langmuir–Hinshelwood mechanism, where both reagents are chemisorbed before the reaction. Through surface analytical and spectroscopic techniques, correlations between solid surface models and the catalysis effect have been investigated, offering an atomic-level understanding of different aspects of heterogeneous catalysis [82,83,84,85]. The behavior of high surface area-supported catalysts is influenced by single-crystal studies, as evidenced by the kinetics of multiple catalytic reactions. Furthermore, three distinct absorption features are shown in the infrared spectra of adsorbed CO on model silica-supported Pd catalysts (where Pd nanoparticles range in diameter from 40 to 500 Å, as confirmed by scanning probe techniques). These features correspond to CO adsorbed onto threefold hollow configurations, with the predominance of these configurations strongly dependent on particle size. The IR absorption features sharpen and become more dominant when the Pd particle size is increased to 100 Å and then to 500 Å. The IR absorption features and the dominance of the peak correspond to the CO adsorbed in the bridging position. The results indicated that larger particles have well-defined crystal orientations whereas the smaller particles have a much more inhomogeneous distribution of CO adsorption sites [86,87].

Another aspect inherent to nanoparticle catalysts is the influence of their morphology on the catalyzed reaction activity. Materials at the nanoscale have very high surface energy and tend to stick to each other. In most cases, nanoparticles are deposited on a substrate in distinct ways, before being used in the reaction. Therefore, the substrate prevents the nanoparticles from aggregating, traps them, and stabilizes them. Particles are said to have large curvatures when at the nanoscale, their surface atoms are unstable. This is particularly evident in particles that have many edge and corner locations. In this case, as the surface-to-volume ratio increases, the catalytic activity increases. This has been confirmed in most research studies using the X-ray diffraction technique and the corresponding observed efficiencies; for example, experimental measurements of CO oxidation activity with supported gold nanoparticles have shown that it is quite independent of the type of substrate chosen, and displays a variation close to *D^−^*^3^, where *D* is the characteristic dimension of the gold nanoparticles [88]. This provides evidence for the dominant influence of the proportion of gold atoms composing the edges of the particles. This effectively contrasts with what is expected from the simplified spherical model of nanoparticles that points to a typical 1/*D* variation.

In addition, Monte Carlo-based algorithms have allowed the simulation of gold clusters ensembles in the particular catalytic size range of 1–6 nm. The results showed that the catalytic activity is dominated by atoms with coordination number 6 or lower, i.e., atoms in a “corner-like” local coordination [89].

The effect of reactant confinement is also an important issue in nanophysics studies of catalysis because chemical reactions generically require that particles come into close contact. Since the reactive collision cross-section dictates the product yields [90], in practice, the reaction is often “imperfect” and may require several random encounters between reactants to achieve significant efficiency.

**Figure 4:**
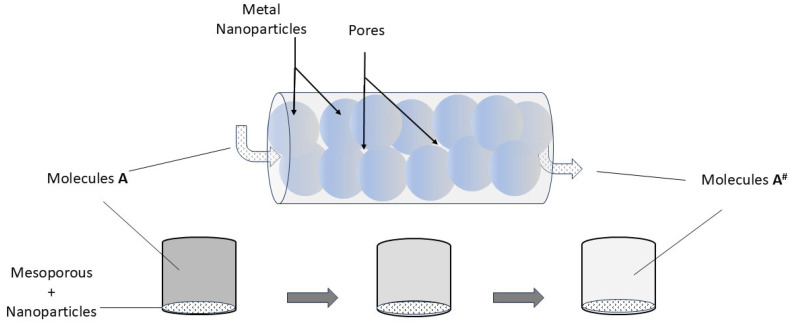
Catalytic activity: pore diameter and electrolyte concentration.

It is thus expected that in confined geometries, the scaling of the collision time with the nanoporous characteristic dimension determines the reaction performance. The collision time, which represents the average time spent by a molecule in collision with another molecule or the surface, is influenced by the confinement size of the pores. In smaller pores, molecules have less space to diffuse before encountering other molecules or the pore walls, leading to shorter collision times. Thus, confinement within nanopores restricts the movement of molecules, effectively reducing their diffusion coefficients. Furthermore, molecules near the pore walls may experience interactions with the surface, such as adsorption or desorption, which affect their mobility and collision dynamics. The collision time may be further influenced depending on the strength and nature of these surface interactions. In addition, the interconnectedness of nanopores within the material can affect the collision time, because it influences the pathways available for molecular diffusion. Highly interconnected pores may facilitate faster diffusion and shorter collision times than with isolated pores. Generally, and theoretically speaking, as pore size decreases in nanoporous materials, the collision time tends to shorten due to increased confinement and reduced diffusion lengths. However, the specific scaling behavior may vary depending on factors such as pore shape, surface chemistry, and the nature of the molecules involved. The confinement effect can lead to enhanced reaction rates, improved selectivity towards desired products, and even the stabilization of reactive intermediates that would otherwise be unstable in bulk solutions [91]. Figure 4 displays a schematic view of the effect of metal nanoparticles/mesoporous porous matrix in a catalytic process.

One concrete example mirrors the importance of endohedral fullerenes in catalysis and their potential application in hydrogenation reactions. Traditionally, these reactions are catalyzed by transition metal catalysts (Pt, Pd, Ni). However, these have high costs, limited selectivity, and susceptibility to poisoning by reaction intermediates. As an alternative, there are the endohedral fullerenes, given their unique electronic and structural properties. Endohedral fullerenes can serve as efficient catalysts for this transformation by providing a confined environment where hydrogen molecules can be activated and selectively transferred to the unsaturated bonds of the substrate. Furthermore, endohedral fullerenes offer advantages such as increased stability, recyclability, and resistance to poisoning [24].

For identical reasons, replacing platinum catalysts with endohedral fullerenes in water electrolysis (both hydrogen evolution reaction (HER) and oxygen evolution reaction (OER)) is a significant goal in the field of renewable energy and sustainable chemistry. Specific endohedral fullerenes designed explicitly for use as catalysts in electrolysis processes are still in the early stages of development. However, some research has explored the potential of La@C82 in oxygen reduction reactions (ORRs), a key process in fuel cells. While not specifically for electrolysis, their catalytic activity could, in principle, be extended to other electrochemical reactions, including OER. Also, endohedral fullerenes containing transition metal atoms, such as Fe, Co, or Ni, while not specifically tailored for electrolysis, could potentially serve as catalysts for HER or OER with further optimization [24]. It is worth mentioning that the synthesis of the chosen endohedral fullerene catalyst should be scalable and reproducible to facilitate large-scale production.

**Figure 5:**
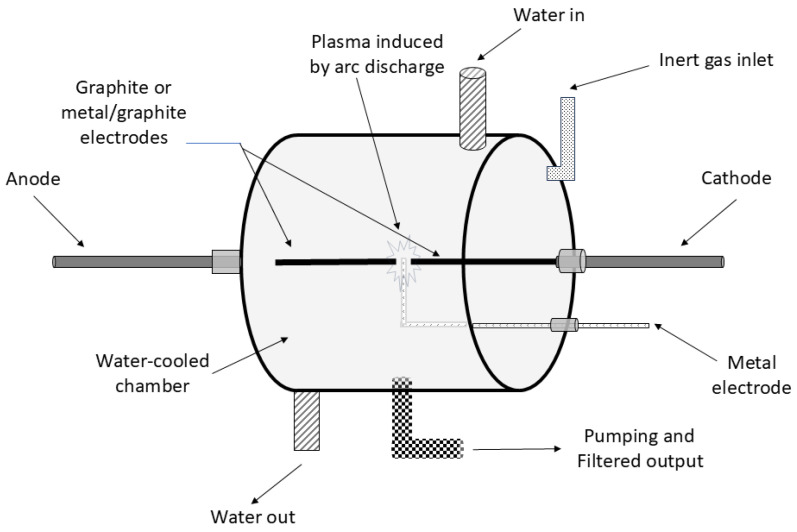
Schematic view of a DC arc discharge apparatus.

Typically, this can be achieved by the Krätschmer–Huffman arc-discharge generator method (Figure 5), the most popular DC arc-discharge apparatus for the large-scale synthesis of endohedral metallofullerenes [92]. Endohedral fullerenes can exist in various redox states depending on the type and the number of metals incorporated. Heterogeneous electron transfer reactions can change this redox state. Studies of the electron transfer at electrodes by cyclic voltammetry and spectro-electrochemistry demonstrated that for La@C_82_, the valency of the metal ion in endohedral fullerenes is caused by the type of the element and not by the shape and redox properties of the carbon cage.

For La@C_82_, it was shown that the charge at the carbon cage is varied by heterogeneous electron transfer and in the higher cathodic region, a new redox state of La inside of La@C_82_ was found [93]. Also using in situ ESR spectro-electrochemistry of La@C_82_, it was shown that the charge at the carbon cage was varied by heterogeneous electron transfer, and a new state of the endohedral La@C_82_ was found in the higher cathodic region.

Modified nanocarbons can also enhance the selectivity and efficiency of catalysts, leading to improved hydrogen production rates. For instance, carbon nanotubes and graphene have been used as electrodes in electrolyzers, where they facilitate efficient water-splitting reactions, leading to improved hydrogen generation rates and energy conversion efficiency [94,95,96,97]. Regarding the domain of hydrogen storage in solid media, several experimental studies have already used modified nanocarbons [97,98,99,100,101].

To end this section about nanophysics in catalysis for clean energy, one needs to dedicate a few considerations to the role that plasmas play in nanocatalysis. Plasmas are considered one of the best possible methods to lead nanophysics and nanotechnology for improving industrial production. Contrary to thermal plasmas where the heavy species temperature is approximately equal to the electron temperature, a cold plasma is not in thermodynamic equilibrium because the electron temperature is much hotter than the temperature of heavy species (ions and neutrals). Cold plasmas usually have lower densities of charged particles than thermal plasma. Once only electrons are thermalized, their Maxwell–Boltzmann velocity distribution differs greatly from the ion velocity distribution. Compared to nonequilibrium plasmas, which use energy very efficiently because electrons can consume the majority of the energy, thermal plasmas are more expensive in terms of energy invested [102]. Because nonequilibrium plasmas have the potential to open chemical pathways that may not be achievable through thermal means, they are more desirable for the synthesis of materials. Furthermore, temperature-sensitive materials can be employed in low-temperature processes. The mechanism for nucleation is as follows: A precursor, such as SiH4, is introduced into the microplasma and through electron impact dissociation, forms reactive radical species, such as SiH3. When the right conditions are met, these radicals can collide, react, and form small clusters. Next, the clusters will agglomerate, or grow, due to more radical or vapor deposition on the particle surface. Finally, the particles will exit the microplasma volume as an aerosol flow.

Plasma-assisted catalysis has several applications in increasing the energy efficiency of several reactions. For instance, in the synthesis of methanol from CH_4_ and CO_2_ in a dielectric barrier discharge, the process is enhanced with the introduction of a catalyst. This way, energy waste and energy costs are reduced. The overall catalyst activity is measured by the conversion, which is the number of moles of reactant converted into products divided by the number of moles of reactant fed into the process. The optimum activity is obtained for an optimum interaction energy between the adsorbates and the catalyst.

In reactive dynamics, the translational energy is more efficient than the vibrational energy in activating early activation barriers (which means that the barrier is encountered before the bond has noticeably elongated). The reverse is true for late activation barriers. It happens that vibrational excitation is important in some plasmas [103]. Thus, the energy amount deposited in vibrational excitations can be tuned to some level. This implies that some of the vibrational energy is available for decreasing the energy barrier to be surmounted, by increasing the reactant energy by a certain amount. Additionally, vibrationally excited species may also experience a lower activation barrier than ground-state species, as ground-state species may not have access to the portion of phase space containing the lowest transition barrier [103]. A typical example of an early-barrier reaction is the dissociative adsorption of H_2_ on transition metal surfaces. In turn, a late-barrier reaction can be exemplified by the dissociative adsorption of H_2_ on noble metals [103].

Because cold plasma is only used sporadically for a small portion of the time needed to saturate the adsorbent catalytic material, plasma catalysis frequently provides benefits over a continuous thermal system, including significant energy savings. Species that are vibrationally excited and interact with catalytic surfaces may also be involved in a nonthermal plasma, apart from electrons. In plasma catalysis, the catalyst influences the discharge, or the plasma interacts with the catalyst. The creation of radicals and excited states, the reduction in active metal, and the modification of the catalyst’s properties are a few examples of how the plasma affects the catalyst; on the other hand, the catalyst’s effects in the plasma include adsorption on the catalyst surface, influence on plasma generation, and packed-bed effect. The energy efficiency is improved by the convergence of all these synergistic effects. When a NiO–Al_2_O_3_ catalyst is processed using atmospheric-pressure methane plasma [104], the low-temperature plasma reduces NiO to Ni (4NiO + CH_4_ → 4Ni + CO_2_ + 2H_2_O); the process is finished when no more CO_2_ evolves. In this instance, the reduction is accomplished at a lower temperature because thermal reduction happens at temperatures above 400 °C. Then, through the fragmentation of adsorbed CH_4_ on the catalyst surface’s active sites to form active adsorbed carbon and hydrogen, the Ni-catalyzed reaction CH_4_ → C + 2H_2_ produces hydrogen with high selectivity.

With nonthermal plasma assisting and argon serving as the discharge gas, an MOF’s behavior during the water–gas shift reaction—which typically only happens at high temperatures—showed exceptional catalytic activity under these circumstances [105]. Enhanced stability is the result of the plasma’s promotion of H_2_O dissociation, which supplies the intermediate OH needed to enable the reaction and stops the organic linker from decomposing due to water. Many MOF plasma-activated experiments demonstrating the synergy of MOF catalysis and nonthermal plasma activation [105] support the promising stability enhancement of MOFs induced by plasma treatment. Hollow nanocages, such as metal-fullerenes or MOFs, can therefore remain stable in the presence of water and under nonthermal plasma activation.

One classic example of how plasma catalysis can work in tandem to produce value-added chemicals is the conversion of CO_2_ in DBD plasma reactors running at atmospheric pressure. The enhanced performance of plasma catalysis can be partially explained by the vibrational excitation of CO_2_ (as well as the co-reactants CH_4_ and H_2_) in the plasma, which facilitates a simpler dissociation on the catalyst at low temperatures. Moreover, the properties of the catalyst (chemical composition or catalytic structure) can be suitably influenced by the plasma electrons. Moreover, it is possible that within the catalyst pores, transient plasma species like excited O atoms could form. Consequently, a catalyst with a high dielectric constant should be preferred since it causes the pellets to polarize more strongly and generates higher electric fields, promoting species dissociation inside the catalyst [103]. In addition to CO_2_ conversion, other processes that combine catalytic and plasma effects, like steam reforming of natural gas, also show synergistic effects. Since this process is a significant source of hydrogen gas, it has drawn special attention.

Nevertheless, there are several drawbacks to the current non-plasma process where water vapor reacts with methane: high-temperature requirements lower the process’s energy efficiency; impurities may eventually cause deactivation; additional hydrogen separation from the syngas product is required; or the process may proceed with a water shift gas reaction that produces carbon dioxide. Because the process is endothermic (165 kJ/mol), a lot of energy is needed. Thus, plasma reforming can offer several advantages, and it was found that the effect of steam addition increased the CO_2_ yield, despite a decrease in methane conversion [106]. The plasma–catalyst synergy effect may be attributed to the presence of vibrationally excited CH_4_ molecules, produced in the plasma [107]. It should also be noted that plasma–catalyst synergy plays a decisive role in the production of CNTs [108].

Operation at a reduced temperature leads to reduced catalyst poisoning and, therefore, increased catalyst lifetime. Moreover, the desirable use of renewable energies in powering plasma reactors helps to ensure full energetic, economic, and environmental sustainability. To reinforce the importance of synergistic effects in plasma and catalysis, one should still note that there are signs that external electric fields are prone to influence the charge polarization on an endohedral fullerene surface [109]. In theory, the polarization of charges on the surface of an endohedral fullerene (or an MOF) due to the encapsulated atom (which can generate dipole moment in the cage) must change the efficiency of its catalytic activity. Using the catalyzed-on-pristine C_60_ hydrogen evolution reaction (HER) as an example, the Gibbs free energy of H^+^ (aq) + e^−^ + ½ H_2_ (g) is too positive to allow H-atoms to adsorb on the surface carbon atoms. However, the H-atom binding free energy on M@C_60_ can be optimized to a perfect value for HER (DG_H_ = 0) when a metal atom M is embedded in the C_60_ cage. Therefore, it is possible to anticipate an increased catalytic activity because of the charge transfer between the metal atom and the C_60_ cage. This may alter the C_60_ cage’s charge distribution, allowing the H-atom to adsorb on M@C_60_. As a result, an external electric field may modify the polarization, which in turn affects the proton adsorption step and the electrocatalytic activity.

## 4. Results and Discussion

There are still challenges to overcoming current setbacks to fully implement the hydrogen economy, mainly regarding efficient production and storage methods. When it comes to this aim, experimental nanophysics makes a decisive contribution to reducing the operating costs of both hydrogen production and storage. The precisely controlled nanoparticle size distribution widths significantly influence critical parameters for both hydrogen production and storage efficiencies. This is accomplished by performing several combined experiments involving the LIP synthesis of nano-electrocatalysts, electrolytic hydrogen uptake, and thermal desorption monitoring. These offer demonstrable benefits in enhancing the electrocatalysis efficiency and the hydrogen storage capacity in solid media. With particular emphasis, one should mention the role of LIP-prepared nanoparticle size distributions in overpotentials’ values. In turn, molecular beam–thermal desorption spectrometry (MB-TDS) allowed the determination of tiny amounts of hydrogen absorbed in a solid medium [110,111]. This is relevant in the search for increasingly efficient hydrogen storage nanomaterials, compared to alternative current methods [111]. Suitable carbon nanostructures produced by LIP also increase the hydrogen storage capacity in the solid state, and carbon-supported catalysts associated with metallic nanoparticles accelerate the hydrogen storage process [112].

To generate carbon nanotubes (CNTs), the LIP process featuring Fe (CO)_5_ vapor flow and ethylene gas was implemented. The fine iron particles produced from the decomposition of organometallic precursor molecules catalyze the formation of CNTs. Ethylene gas is directed through a bubbler containing liquid iron pentacarbonyl at room temperature as a carrier for the Fe (CO)_5_ vapor. This mixture is pyrolyzed in the flow reactor due to the infrared irradiation, which heats the gas. Ethylene gas also serves as a sensitizer, activating the laser reaction and speeding up the Fe (CO)_5_ dissociation. In turn, iron nanoparticles, necessary for catalyzing the formation of CNTs, are obtained from the decomposition of Fe (CO)_5_ during laser-induced reaction [44]. Ethylene has resonant absorption at a wavelength of 10.6 μm and high dissociation energy, which results in internal energy exchange with acetylene molecules that do not absorb the CO_2_ laser radiation, heating the entire gas mixture [45].

**Figure 6:**
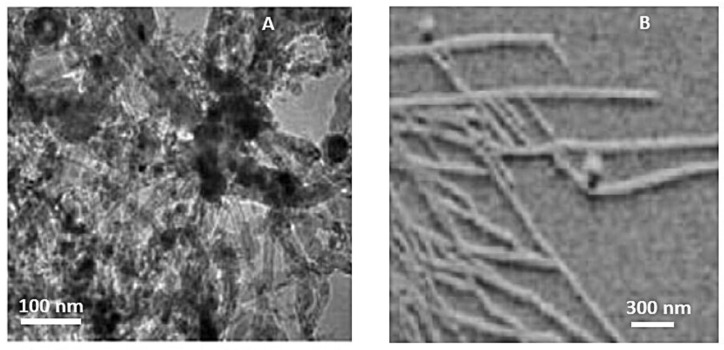
Synthesized powders consisting of CNTs with different structures observed by (**A**) Scanning Electron Microscopy and (**B**) Atomic Force Microscopy (on mica substrate).

The powder obtained (through pyrolysis and incomplete combustion of the carbon precursor) is trapped in the collector’s filter and the soot is weighed, which allows us to estimate the soot production (g/h) and the efficiency (%). For a pressure of 650 mbar, the productivity is around 2 g/h. The as-synthesized powders are investigated by scanning electron microscopy and atomic force microscopy (on mica substrate) in tapping mode. Carbon nanotubes with different structures were indeed observed (Figure 6).

Some nanotubes come together with iron particles, which may have played a role in the catalysis of the nanotube growth [43,44,45]. Such fine iron particles were produced in the same experiment through the decomposition of the organometallic precursor molecules [44]. Specific conditions, such as laser parameters, gas composition, and reaction time, were found to influence the morphology of the soot observed by ultramicroscopy. The laser-induced pyrolysis of the gaseous mixture leads to the formation of both carbon nano-powders and CNTs, depending on the specific conditions employed during the process. This technique offers a possibility for achieving improved control of the uniformity and sharpness of nanocarbons size distributions [46].

**Figure 7:**
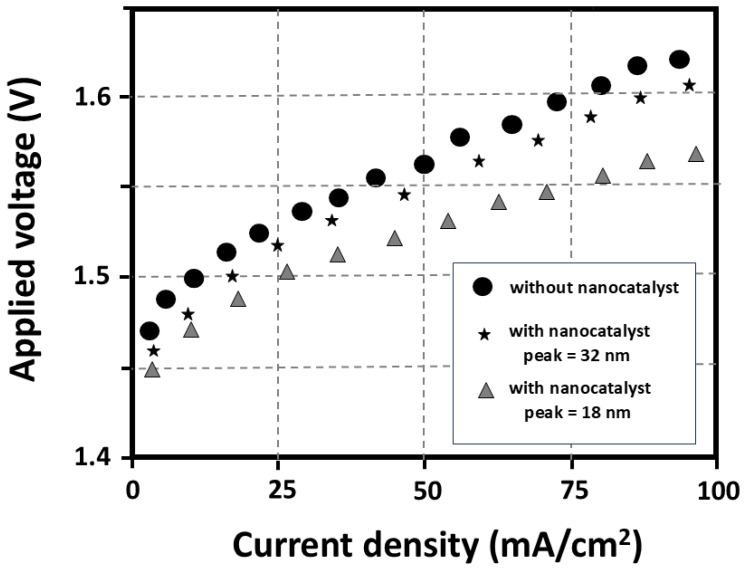
Alkaline water electrolysis with and without nanocatalyst.

The introduction of this iron-doped nanocarbon soot as a nanocatalyst gives rise to an efficiency improvement of the alkaline water electrolysis, confirmed by our experimental data displayed in Figure 7. Green catalytic electrolysis offers convenient scalability to meet energy demands, and it is prone to reduce energy costs by increasing the electrolyzer efficiencies. Moreover, electrolysis can achieve higher energy conversion efficiencies than photoelectrochemical catalytic water splitting (above 80%, against 5% to 20%). Reducing the energy needed to split the water molecules and increasing the surface area available for the reaction will result in appropriate efficiency. Thus, it could be effectively obtained using adequate nanocatalysts, thanks to the unprecedented control over their size. Applying the common relevant parameters of alkaline electrolysis experiments, for the cases “with and without nanocatalyst”, one can draw some conclusions from the estimations of the respective Faradic efficiencies; 75%, 85%, and 90% are the corresponding values obtained for the Faradic efficiencies, respectively, in the situations “without”, “with 32 nm”, and “with 18 nm”. This proves the critical role that nanocatalyst size particle distributions play in the values of the overpotentials. Such improvement can be attributed to the following causes: enhanced electrocatalytic activity assured by the combined iron-carbon nanotube synergic effect, modification of the surface area and surface charge, and conductivity increase (which allows a better electron transfer during electrochemical reactions and a reduction in the extra energy required to drive a reaction: overpotential).

In general terms, it is expected that there is an optimal particle size range for achieving the lowest overpotential values. Extremely small nanoparticles may suffer from increased surface energy, and agglomeration tendencies, which can limit their stability and catalytic performance. On the other hand, excessively large particles may reduce the surface area and limit access to reactants, leading to higher overpotentials. Catalyst nanoparticles in this optimal range offer real advantages. They provide a high surface area-to-volume ratio, maximizing the active site number available for the catalytic reaction. This increased surface area allows for efficient utilization of the catalyst material and enhances reaction kinetics.

The results of Figure 7 allow us to stress that LIP studies in conjunction with Atomic Force Microscopy (AFM) analysis are excellent experimental methods that must be pursued. These outputs deserve more studies regarding the influence of nanocatalyst size distribution; a size range selection that gives rise to the lowest overpotential and highest catalytic activity, while maintaining stability, justifies further research. Effectively, the maxima of the two nanocatalyst size distributions (respectively 18 nm and 32 nm), mirror the decrease in the overpotential with the size decrease. These results prove that LIP gives rise to nanocatalysts with sharp size distributions, and AFM assures a precise measurement of such distributions. Overall, a catalyst influences the polarization curve of water electrolysis by lowering activation energy, enhancing reaction rates, shifting overpotential, and improving selectivity, thereby improving the efficiency and performance of the electrolysis process. A good catalyst for alkaline water electrolysis should indeed give rise to a low overpotential in the polarization curve (in agreement with the results of Figure 7). Reducing the overpotential is important because it directly affects the energy efficiency of the electrolysis process. A lower overpotential allows for a higher current density at a given voltage, which means the electrolysis reaction proceeds more efficiently. This leads to reduced energy consumption and cost.

It is worth mentioning that density functional theory points out that an introduction of a metal dopant can synergistically optimize the electronic structure of CNTs and adsorption-free energy of H-atoms on CNTs, thus promoting the hydrogen evolution reaction [47]. Furthermore, electrocatalytic water splitting suffers from sluggish anodic oxygen evolution reaction kinetics leading to low energy conversion efficiency. Iron encapsulated in doped carbon nanotubes possesses superior hydrogen evolution reaction activity requiring an onset overpotential close to that of 20 wt% Pt/C, which means that it is a small overpotential for a non-precious-metal catalyst. Moreover, doped CNTs can exhibit improved stability and durability compared to pristine carbon nanotubes (note that water electrolysis is often conducted under harsh conditions).

The LIP technique was also used to prepare nickel-doped carbon nanotubes. The procedure is like the one already mentioned, but this time it uses nickel tetracarbonyl, Ni (CO)_4_, instead of Fe (CO)_5_. Additionally, a study of the temperature desorption dependence of hydrogen was performed, which is significant since the adsorption capacity rapidly decreases with the increase in temperature. Surface- or interface-related sites take on critical importance in tiny systems and can alter the overall solubility of hydrogen. Hence, a focus has been placed on thermal desorption research and calibration to improve the kinetic properties of hydrogen storage in nanomaterials. In contrast to the determination of static sorption isotherms, thermal desorption spectrometry ensures fair accuracy in monitoring hydrogen absorbed in solid materials and is ideal for monitoring hydrogen evolution after applying a thermal ramp [113,114].

**Figure 8:**
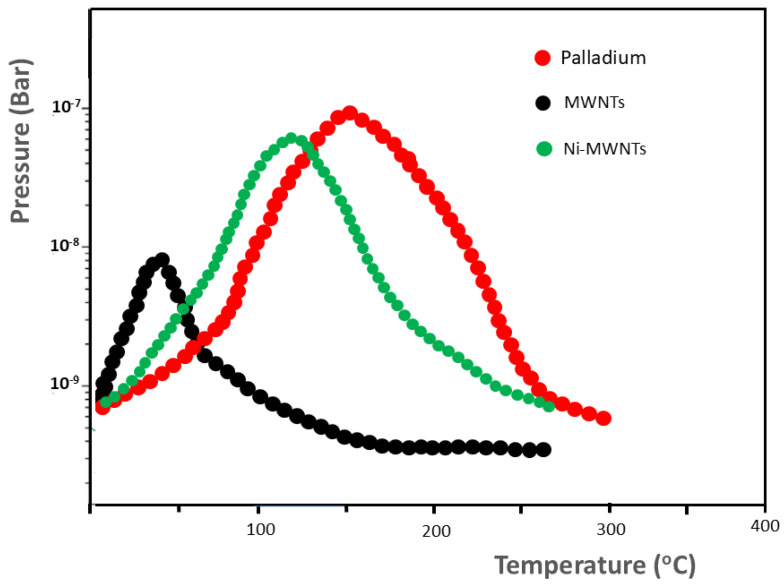
Molecular beam–thermal desorption spectra for the following hydrogen storage solids: palladium; MWNT assembly; Ni-doped CNT assembly.

The identification of developed gas species is made possible by mass spectrometry, and the acquisition data on desorption temperatures provide information on the binding energy of adsorbed molecules, which changes depending on the type of adsorbate/surface material. The molecular beam–thermal desorption spectrometry (MB-TDS) method has been shown to be accurate [72,110], and it is used here for the desorption analysis of a Ni-doped CNT electrode. This electrode was previously submitted to hydrogen electrochemical uptake followed by desiccation under vacuum. It was assembled from LIP-prepared Ni-doped CNT powder. The hydrogen pre-charging was galvanostatically performed, using pure platinum as the anode of an electrolytic cell (where the anode is at each turn made of pure platinum, MWNT assembly, and Ni-doped CNT assembly), containing an aqueous 1 M KOH electrolyte and imposing an electric current of 133 mA. Identical electrochemical hydrogen uptake procedures were applied to the three samples, using the same relevant physical operating parameters. Before and after the electrochemical hydrogen uptake, a Kern ABT-101 analytical balance was used to weigh all the samples. This way, relative weight increases were recorded: 8% for Pd, 4% for the MWNT assembly, and 6% for the Ni-doped CNT assembly. Figure 8 displays the MB-TDS desorption experimental spectra obtained for the three hydrogenated sample electrodes (palladium, MWNT assembly, and Ni-doped CNT assembly), applying the same heating rate (1 °C min^−1^). From the relative corrected area of the spectra given in Figure 8, it is found that they reasonably agree with the expected relative weight ordering.

Therefore, the inclusion of the nickel nanoparticles in the CNT assembly led to a storage efficiency much closer to the ideal pure palladium ability. The CNT’s adsorption capacity appears to be positively correlated with its specific surface area and micropore volume. The recorded MB-TDS sequence can be interpreted through this argument. Specifically, the MWNT assembly has a high specific surface area, and the addition of nickel nanoparticles seems to introduce a synergistic effect that enhances hydrogen absorption. Thus, carbonaceous materials displaying a mesoporous structure can absorb hydrogen through atom insertion, which favors stabilization. When hydrogen gas molecules contact the metal catalyst, they can break apart. Some of the hydrogen atoms stay attached to the metal, while others move through diffusion to the carbon nanotubes.

Controlling the size distribution of nanocatalysts allows for optimization of their surface area, electronic properties, active site distribution, stability, and reaction selectivity, all of which contribute to higher catalytic efficiency. Therefore, this section would not be complete without summarizing how the LIP nanophysics technique is decisive in controlling the size distribution of nanocatalysts, which is crucial for enhancing their catalytic efficiency, since nanocatalyst performance is highly dependent on their size, shape, and surface characteristics. Skipping formulary details, it is easy to understand that if the size distribution is too broad, some nanoparticles might be too large, reducing the total surface area, while others might be too small and less stable.

Reaction rates are increased by maximizing surface atom exposure throughout the catalyst by maintaining a narrow size distribution. Consistent catalytic performance results from a narrow size distribution, which guarantees that all nanocatalysts display comparable electronic characteristics. Variability could be introduced by a wide distribution, which would lower overall efficiency. It is possible to adjust the reaction kinetics to optimize speed and stability over time by preserving a consistent size distribution. A greater percentage of surface atoms are exposed by smaller nanoparticles; however, if the size distribution is wide, larger particles will predominate, lowering the effective catalytic surface. By regulating the PSD, one can prevent extremes where too-small particles might show undesired quantum effects and guarantee that all particles similarly contribute to catalysis. Furthermore, a broad size distribution may lead to variability in the reaction rate constant. Larger nanoparticles may experience slow mass transfer, which lowers catalytic efficiency, but smaller nanoparticles, with a higher surface-to-volume ratio, lessen diffusion limitations in terms of mass transfer. Therefore, optimal diffusion is assured, and diffusion limitations are minimized by a well-controlled size distribution.

In summary, by ensuring a narrow size distribution, catalytic efficiency is maximized by optimizing the surface area, electronic properties, reaction kinetics, and stability.

For hydrogen production and storage applications, it is crucial to understand how various LIP experimental parameters and conditions influence the particle size distribution (PSD) of the synthesized materials. The width of this distribution has a direct effect on the efficiency of the material in hydrogen storage and hydrogen production. The following are the key factors that need to be carefully controlled or optimized to influence the PSD: The laser wavelength dictates the energy coupling with the precursor molecules, influencing the particle formation dynamics, and the laser power determines the temperature in the reaction zone. Higher power increases the temperature in the reaction zone, leading to a more complete decomposition of precursor molecules and promoting the formation of smaller, more uniform particles. However, too high a power can cause excessive heating and vaporization, leading to a wider particle size distribution due to non-uniform nucleation and growth processes. In turn, the laser spot size at the focal point imposes the volume of the reaction zone. A smaller spot size concentrates energy, leading to more localized heating and faster pyrolysis, which could lead to a broader PSD due to non-uniform cooling in the immediate reaction zone. Conversely, a larger focus might lead to more uniform particle growth due to a more consistent temperature profile.

The quenching rate refers to how rapidly the reaction products are cooled after nucleation. Rapid quenching leads to a narrow PSD because the particles have less time to grow through agglomeration or sintering. Quenching can be controlled by adjusting the carrier gas flow rate, chamber cooling systems, or introducing external quenching agents like cold gasses or liquids. Insufficient quenching leads to a broader PSD due to extended particle growth. Regarding the carrier gas, its flow rate dictates how quickly the products are quenched and transported out of the laser reaction zone. Faster flow rates remove particles more rapidly, minimizing particle growth after nucleation and leading to narrower PSDs. Slower flow rates allow more growth and can lead to broader PSDs as the particles have more time to aggregate and grow.

## 5. Conclusions and Remarks

In the current worldwide context, implementing a sustainable energy system is one of the most important industrial challenges. Hydrogen energy is considered the most powerful candidate to replace fossil energy due to its clean, renewable, and environmentally friendly properties and high energy density. Electrocatalytic green hydrogen evolution associated with suitable safe transportation methods grounds important ways to develop a sustainable energy system based on hydrogen technologies.

The benefits of nanotechnology in the hydrogen-based energy transition are explained here on a real experimental basis. The high hydrogen adsorption capabilities of the nanomaterials used in this work satisfy the criteria of having a high surface area, tailored pore size and shape, high storage capacity, controlled desorption, and safety. Laser-induced pyrolysis adds the advantage of producing interesting sorts of very clean nanocatalysts and nanomaterials from nanoparticles with a narrow size distribution. The amount of metal incorporation can be controlled by adjusting the corresponding precursor’s concentration (in this work’s case, iron pentacarbonyl, or nickel tetracarbonyl) or the growth conditions. Experimental results prove the critical role that nanocatalyst size particle distributions play in the values of the overpotentials. A Faraday efficiency of 90% could be achieved using an 18 nm peak iron-doped mixed nanocarbon/CNT size distribution, which means that 0.55 moles of hydrogen are produced for every mole of water. This undoubtedly reflects the importance of controlling the width of the nanoparticle size distributions. It is also demonstrated that carbon nanotubes combine their intrinsic advantages (low density, high mechanical resistance, chemical inertia, and compactness) with most of the adsorbed hydrogen being released at convenient conditions of pressure and temperature.

High hydrogen pressure and cryogenic temperatures are not necessary when storing hydrogen in the right solids, which can result in a safer and more compact method. The use of hydrogen storage solids as active materials in fuel cells has been the subject of numerous studies. Numerous high-surface nanoporous materials have been studied, primarily classified into two groups, based on carbon and MOFs [115]. Because only a small portion of the pores in activated carbons’ pore-size distributions are small enough to interact strongly with hydrogen molecules in the gas phase, activated carbons are ineffective in hydrogen storage systems. Surface- or interface-related sites become extremely important in small-scale systems and can modify the hydrogen’s overall solubility.

The solid-state hydrogen storage capacity can be enhanced by appropriate carbon nanostructures, and the hydrogen storage process can be sped up by carbon-supported catalysts containing metallic nanoparticles. Thus, this explanation of the advantages of nanotechnology in the hydrogen-based energy transition is based on actual experimental data. High hydrogen adsorption capabilities can be ensured by using nanomaterials that meet the requirements of having a high surface area, customized pore size and shape, high storage capacity, controlled desorption, and safety. The benefit of creating intriguing, ultra-clean nanocatalysts and nanomaterials from nanoparticles with a narrow size distribution is enhanced by laser-induced pyrolysis. By modifying the precursor (iron pentacarbonyl or nickel tetracarbonyl) concentration or the growth conditions, the amount of metal incorporation (Fe or Ni) can be regulated.

It has been demonstrated that the amount of adsorbed hydrogen released (at a comfortable pressure and temperature) combines with the intrinsic advantages of carbon nanotubes (low density, high mechanical strength, chemical inertness, and compactness). Using the MB-TDS technique, a comparative experimental study was carried out on advanced carbon nanostructured electrodes under conditions like hydrogen uptake/desorption. A positive correlation between CNT’s hydrogen adsorption capacity and its specific surface area/microporosity, possibly due to a synergistic effect caused by the addition of nickel nanoparticles, could be experimentally verified.

In conclusion, some say that in materials designed for hydrogen storage, a narrow PSD is desirable for maximizing the surface area and ensuring uniform adsorption/desorption kinetics. Smaller, uniform particles increase the surface area-to-volume ratio, improving hydrogen uptake and release rates. For materials used in hydrogen production via processes like water splitting, the PSD influences the surface reaction kinetics. Narrow PSDs in photocatalysts like TiO₂ or MoS₂ ensure uniform photon absorption and charge carrier dynamics, leading to higher catalytic efficiency. A broader PSD, on the other hand, may introduce non-uniform light absorption and diffusion limitations, reducing the overall efficiency of hydrogen production.

The key performance parameters in alkaline hydrogen electrolysis are the electrochemically active surface area (ECSA), the current density, and the overpotential. The ECSA is critical for the efficiency of electrolysis. Smaller particles provide a larger surface area for the same total mass of catalyst, increasing the number of active sites for the hydrogen evolution reaction (HER) and oxygen evolution reaction (OER). The current density is proportional to the number of active sites, which in turn is proportional to the ECSA. Therefore, the current density is also inversely proportional to the average particle size, assuming a narrow PSD. Larger particles (or a broader distribution) result in fewer active sites and a lower current density. The overpotential is the extra voltage required beyond the thermodynamic potential to drive the electrochemical reaction. A larger surface area leads to lower overpotentials, increasing the energy efficiency of the process.

Hydrogen storage efficiency is often correlated with the rate at which hydrogen is absorbed/desorbed, which in turn is influenced by both the surface area of the nanocatalyst particles and their size distribution. Thus, a narrow PSD leads to more efficient hydrogen absorption and desorption, improving the overall performance of nanocatalysts.

## Data Availability

Not applicable.
